# Safety and efficacy revisited: a systematic review and meta-analysis of glue versus tack mesh fixation in laparoscopic inguinal herniorrhaphy

**DOI:** 10.3389/fsurg.2024.1321325

**Published:** 2024-02-09

**Authors:** Sandesh Raja, Adarsh Raja, Ziyan Ansari, Sara Eman, Simran Bajaj, Muhammad Ahmed, Uday Kumar, Yawar Hussain Shah, Sachin Jawahar, Faisal Aftab, Deepak Rajani, Satesh Kumar, Mahima Khatri

**Affiliations:** ^1^ Department of Surgery, Dow Medical College, Dow University of Health Sciences, Karachi, Pakistan; ^2^ Department of Surgery, Shaheed Mohtarma Benazir Bhutto Medical College Lyari, Karachi, Pakistan; ^3^ Department of Surgery, Foundation University Medical College, Islamabad, Pakistan; ^4^ Department of Surgery, Shaheed Mohtarma Benazir Bhutto Medical University, Larkana, Pakistan; ^5^ Department of Surgery, Liaquat College of Medicine and Dentistry, Karachi, Pakistan

**Keywords:** inguinal hernia, laparoscopic herniorrhaphy, glue mesh fixation, tack mesh fixation, hernia

## Abstract

**Background:**

This analysis addresses the uncertainty surrounding the efficacy of glue mesh fixation (GMF) compared with tack mesh fixation (TMF) in laparoscopic herniorrhaphy. Our meta-analysis incorporates recently conducted randomized controlled trials (RCTs) to enhance the reference for assessing the efficacy and safety of GMF.

**Methods:**

PubMed Central, Google Scholar, Science Direct, and Cochrane Library were extensively reviewed for articles in the English language performed from inception to May 2023 using the keywords “Glue mesh repair,” “Tack mesh repair,” “Inguinal Hernia,” “Herniorrhaphy,” “Laparoscopic,” “Mesh Fixation,” and “Randomized controlled trials.”

**Results:**

In this meta-analysis, we incorporated a total of 20 randomized controlled trials, evaluating each article individually using quality ratings. Compared with TMF, GMF demonstrated a significant reduction in the incidence of chronic pain [RR: 0.40, (0.23, 0.68)] and pain scores on postoperative day 1 [MD: −1.07, (−1.90, −0.25)]. We also used funnel plots and Egger's regression to test for publication bias.

**Conclusion:**

In summary, this meta-analysis establishes the significance of GMF in reducing chronic pain and postoperative day 1 pain compared with TMF. However, no statistically significant difference was noted between the GMF and TMF groups concerning hematoma, seroma, operation time, recurrence rate, and total complications. Nonetheless, given the small number of cases in this study, the findings must be validated in the future by multicenter, large-sample, high-quality RCTs.

## Introduction

Hernia manifests when tissue protrudes beyond its anatomical confines. The bulge is most noticeable while standing, coughing, or straining. Obesity, pregnancy, hard lifting, COPD, and aging can all cause it to be congenital or develop later in life (1). Hernias are often diagnosed clinically and confirmed with imaging such as ultrasound and MRI (2). Herniorrhaphy, a popular surgical treatment, is used to correct approximately 800,000 inguinal hernias in the United States each year (3). A crucial reason for urgent hernia therapy is strangulation, which causes a loss of blood flow to trapped tissue (4, 5). Inguinal hernias constitute more than 75% of all abdominal hernias and are predominantly found in men, making them a frequently encountered medical condition by general surgeons (6, 7). There is a greater chance of discomfort, tissue damage, and nerve entrapment when employing open surgical techniques. Consequently, it is preferable to use less invasive laparoscopic techniques such as transabdominal preperitoneal (TAPP) and completely extraperitoneal (TEP) techniques (8–10). While the TAPP method entails dissecting the abdominal wall, which increases the risk of gastrointestinal injuries, the TEP approach bypasses the abdominal cavity, preventing injury and adhesions (11). The most frequent consequence, inguinodynia (pain following surgery that lasts longer than 3 months) (12), influences surgeons' decisions between adhesive mesh and penetrative tacker techniques (13, 14).

Glue mesh fixation (GMF), compared with tack mesh fixation (TMF) provides less tension on the surrounding tissue (15). The exploration of mesh fixation with fibrin glue or cyanoacrylate tissue adhesive, as an alternative to traditional suture or tack methods, has shown remarkable results (16). The study conducted by Nizam et al. (17) demonstrated that the fibrin glue method proved to be a cost-effective approach that resulted in a reduced hospital stay compared with the TMF group. The GMF method has also reduced the risk of inguinodynia and recurrence in randomized controlled trials (RCTs) over comparative groups (18, 19).

In a prior meta-analysis conducted by Nan Hu (20) comparing GMF and TMF, it was concluded that GMF is more efficacious in diminishing the incidence of chronic pain compared with TMF. Moreover, the findings suggest a lower occurrence of hematoma in the GMF group compared with the TMF group. Notably, this meta-analysis does not reveal any disparities in the pain score on postoperative day 1, operation time, and recurrence rate between the GMF and TMF groups. Conversely, the analysis highlights a significant distinction, indicating that GMF markedly reduces the risk of total complications. Certain outcomes in the analysis were assessed using a fixed-effects model; however, it is recommended to utilize a random-effects model when incorporating studies with diverse effect sizes, as most of the studies included in this analysis consist of varied effect sizes (21).

There is only a slight variation between these two methods and requires further assessment. In this meta-analysis, we aim to address a gap in the current body of research by conducting a thorough examination of the available RCTs that investigate the impact of glue mesh vs. tack mesh in patients undergoing laparoscopic inguinal hernia repair. Our study's main objective is to conduct a thorough analysis of primary outcomes, such as chronic pain and pain on postoperative day 1, and secondary outcomes, including recurrence rate, total complications, hematoma, and seroma.

## Methods

This meta-analysis was conducted by following Preferred Reporting Items for Systematic Review and Meta-Analysis (PRISMA) guidelines (22).

### Study selection

A literature search was conducted on PubMed Central, Google Scholar, Cochrane Library, and Science Direct from inception to May 2023. The search strings used in different databases are given in Supplementary Table S1. All duplicated articles were removed using Endnote ×9 (Clarivate Analytics, USA). Two separate individuals (MA and YS) carefully reviewed the remaining articles and selected articles to be analyzed that matched the inclusion criteria mentioned below. Articles were selected based on the title/abstract, and then a full-text evaluation was conducted. In case of disagreement, a third reviewer (AR) was consulted. Articles were selected based on the following eligibility criteria: (a) studies comparing GMF vs. TMF using a laparoscopic technique, (b) patients of age 18 years or above with inguinal hernia, (c) studies with at least one outcome of interest, (d) randomized controlled trials. The outcomes of interest were chronic pain, which was defined as persistent pain for more than 3 months, pain score on postoperative day 1, operation time, recurrence rate, which refers to the number of cases in which hernia recurred after the initial surgical intervention, seroma, hematoma, and total complications. Any non-human trial, language apart from English, patients under the age of 18 years, suture fixation method and open repair techniques, duplicated studies, articles irrelevant to the research purpose, and studies with incomplete information regarding outcomes were excluded.

### Data extraction and quality assessment

In each study, the following data were extracted: (a) study name and year, (b) the number of patients in each group (TMF vs. GMF), (c) general patient characteristics (age, gender, and BMI), (d) follow-up time, (e) type of approach used (TAPP or TEP), (f) type of tacks and glue used, and (g) outcomes of interest. The quality assessment of the selected RCTs was conducted independently by two reviewers (AR and SA) using Cochrane risk of bias (RoB 2.0) tools including (1) random sequence generation (selection bias), (2) allocation concealment (selection bias), (3) blinding of participants and personnel (performance bias), (4) blinding outcome assessment (detection bias), (5) incomplete outcome data (reporting bias), and other bias. Each was assessed individually, and the potential risk for each outcome was characterized into three groups: low risk, high risk, or unclear (23).

### Statistical analysis

The statistical analysis utilized Review Manager (RevMan Version 5.4.1), which is a software provided by the Cochrane Collaboration Network. Dichotomous data were used to derive the risk ratio (RR) and corresponding 95% confidence intervals (95% CIs). Similarly, for continuous outcomes, the mean difference (MD) and their 95% CIs were obtained using a random-effects model. A *p*-value of less than 0.05 was judged as significant. Higgins *I*^2^ was used to measure heterogeneity. The value of *I*^2^ = 25%–50% was regarded as mild heterogeneity, 50%–75% as moderate, and >75% as high heterogeneity (24). Funnel plots were created for the outcomes that included more than 10 studies to check for any publication bias. The Egger test was performed to check if there were any publication bias. Continuous outcomes reported as median with interquartile ranges were converted to mean and standard deviations using Wan's method (25).

## Results

### Study selection and characteristics

**Figure 1:**
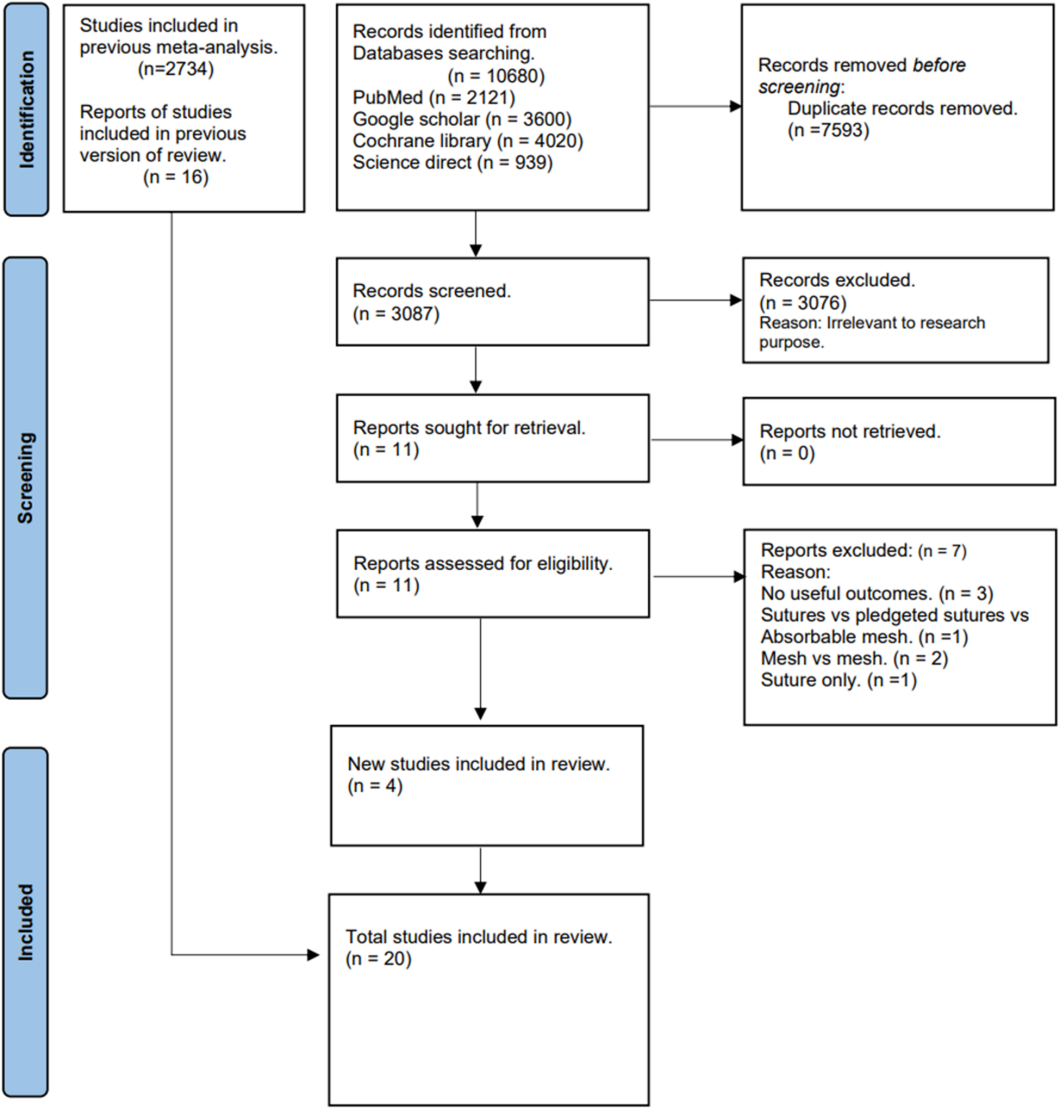
PRISMA flow chart.

After eliminating duplicates, our initial literature search yielded 3,087 relevant articles. Following the screening of titles and abstracts, 11 articles were assessed for eligibility, resulting in the exclusion of seven articles (26–32). The reasons for exclusion are outlined in Figures 1, 4. Subsequently, four additional studies (15, 19, 33, 34) were incorporated into the synthesis, along with 16 studies from previous meta-analyses (17, 35–49), particularly the one conducted by Nan Hu et al. (20). In total, this meta-analysis included a total of 20 articles (15, 17, 19, 33–49). The collective sample involves 2,928 patients with inguinal hernias, distributed as 1,582 in the TMF group and 1,346 in the GMF group, meeting the inclusion and exclusion criteria. The mean age of patients in the TMF group was 51.75 years, and in the GMF group, it was 51.39 years, with an average follow-up time of 12 months. The PRISMA flow chart, depicted in Figure 1, provides a concise overview of the outcomes derived from our extensive literature review. In addition, Table 1 furnishes the baseline characteristics of patients in each study.

**Table 1:** Baseline characteristics of studies.

		Participants (*N*)		Mean age (SD)		Mean BMI (SD)			Intervention			
Author, year	Patient population (*N*)	Tacker mesh (*n*)	Glue mesh (*n*)	TMFG	GMFG	TMFG	GMFG	Type of operation	Type of staple	Type of glue	Type of mesh	Follow-up (months)
Boldo et al, (36)	22	11	11	57.7 (12.8)	57.7 (12.8)	25.4 (2.6)	25.4 (2.6)	TAPP	staples ProTack device (USSC Auto Suture, Norwalk, CT, USA) was used in the SG	autologous fibrin sealant (Vivostat system)	mesh	6 m
Brügger et al, (37)	77	40	37	59.9 (19.9–82.2)	57.3 (20.9–82.5)	24.7 (18.4–32.6)	24.8 (20.9–33.7)	TAPP	ProTak device (5 mm titanium)	Glubran cyanoacrylate tissue sealant	Vypro II prosthetic mesh (10 cm^2^ × 15 cm^2^)	38 m
Bunkar et al, (38)	60	30	30	48.77 (13.19)	49.63 (16.89)	23.6 (3.2)	23.89 (3.4)	TEP	(ProTack 5-mm fixation device	n-butyl 2-cyanoacrylate (NBCA) glue mesh fixation	mesh	6 m
Chandra et al, (39)	100	50	50	40.64 (8.39)	41.7 (8.51)	28.72 (4.52)	28.92 (4.66)	TEP	Staples	fibrin glue	mesh	3 m
Cristaudo et al, (34)	146	151	81	_	_	_	_	TEP	Absorbable Tacks	Tisseel Fibrin glue	1.the non-absorbable anatomical mesh 2.non-absorbable folding slit mesh 3.partially absorbable mesh 4. non-absorbable anatomical mesh	3 m
Fortelny et al, (40)	89	45	44	45.0 (14.0)	45.5 (11.3)	25.6 (3.4)	26 (7.2)	TEP/TAPP both	Staples	Tisseel Fibrin glue	macroporous mesh	12 m
Habeeb et al, (41)	532	266	266	_	_	_	_	TAPP	Tacks	Cyanoacrylic tissue glues	polypropylene (10 cm × 15 cm)	18 m
Issa et al, (42)	106	55	51	57.9 (15.2)	48.5 (14.0)	_	_	TAPP	AbsorbaTacks (Medtronic)	Glubran 2 Cyanoacrylate glue	Bard 3D or Parietex anatomical mesh	6 m
Lau et al, (43)	93	47	46	66 (55.0–76.0)	64 (55.8–71.3)	_	_	TEP	Staples (Titanium)	TISSEEL VH 2 ml	Prolene meshes (15 cm × 10 cm)	12 m
Liew et al, (35)	66	34	32	_	_	_	_	TEP	titanium tacks (ProTack-5 mm fixation device)	0.5 ml enbucrilate glue	Prolene meshes (15 cm × 10 cm)	3 m
Lovisetto et al, (44)	197	98	99	53.2 (12.6)	52.9 (14.6)	_	_	TAPP	Endopath Multifeed Stapler 10 mm shaft titanium staples	tisseel fibrin glue	monofilament polypropylene mesh with large pores 10 × 13	12 m
Melissa et al, (45)	129	65	64	53.31 (11.78)	52.77 (10.25)	_	_	TEP	Tack	FS spray (Tisseel; Baxter Healthcare, Deerfield, IL)	10 cm^2^ × 15 cm^2^ lightweight Prolene mesh	6 m
Moreno-Egea, (46)	106	54	52	54.9 (15.6)	55.8 (13.8)	_	_	TEP	2 tacks	n-hexyl-a-cyanoacrylate	10 cm × 15 cm polypropylene mesh	24 m
Nizam et al, (17)	60	30	30	-	-	20.1–25	20.1–25	TEP	tackers	fibrin glue	mesh	3 m
Olmi et al, (47)	600	450	150	44.5 (18–77)	44 (18–77)	_	_	TAPP	EMS, Protak, EndoANCHOR	Tissucol fixation	L-shaped 14–13-cm meshes.	1 m
Subwongcharoen et al, (33)	60	30	30	48.27 (17.33)	52.40 (14.95)	_	_	TEP	staples (ProTack)	Histoacryl (N-butyl-2-cyanoacrylate)	Ultrapro mesh (Ethicon, inc. Johnson-Johnson com) of 13 cm × 10cm	12 m
Tolver et al, (48)	100	50	50	49 (21–73)	50 (29–77)	25 (20–31)	25 (21–33)	TAPP	Tacks titanium	Tisseel Fibrin Glue	Ethicon Ultrapro mesh, 15 cm × 10 cm	6 m
Wasim et al, (49)	60	30	30	_	_	_	_	TEP (30)/TAPP (30)	Tackers	Tisseel Fibrin Glue	intraperitoneal only mesh	24 m
Azevedo et al, (19)	42	21	21	_	_	_	_	TAPP	Absorbatack stapler (Covidien-Medtronic)	N-Butyl-2-Cyanoacrylate (Glubran 2)	heavyweight polypropylene mesh 15 cm × 12 cm	24 m
Jeroukhimov et al, (15)	208	106	102	54.5 (16.0)	54.5 (16.3)	_	_	TEP	absorbable tackers SECURE STRAP	LIQUIBAND FIX 8	knitted polypropylene mesh	12 m

RCT, randomized control trial; TMFG, tacker mesh fixation group; GMFG, glue mesh fixation group; BMI, body mass index; SD, standard deviation; TEP, laparoscopic totally extraperitoneal; TAPP, transabdominal preperitoneal.

### Risk of bias of the included studies

**Figure 2:**
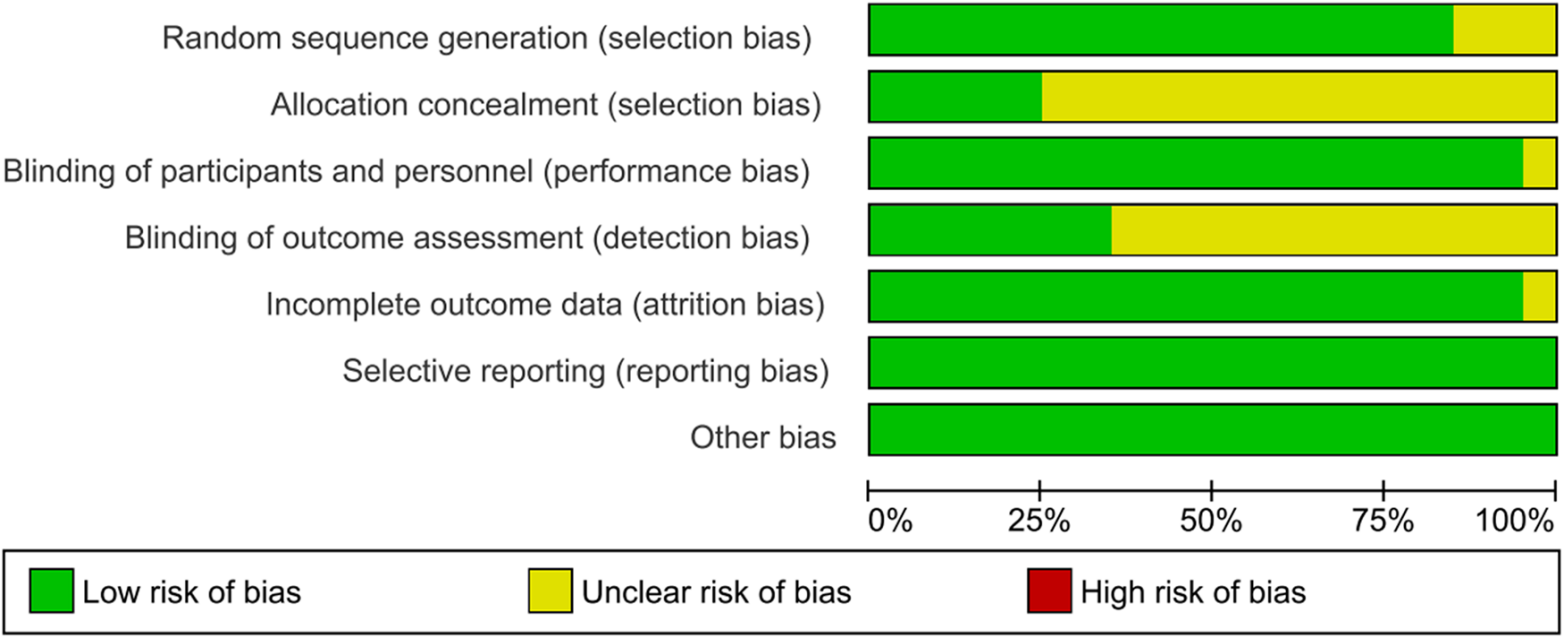
Risk of bias graph.

**Figure 3:**
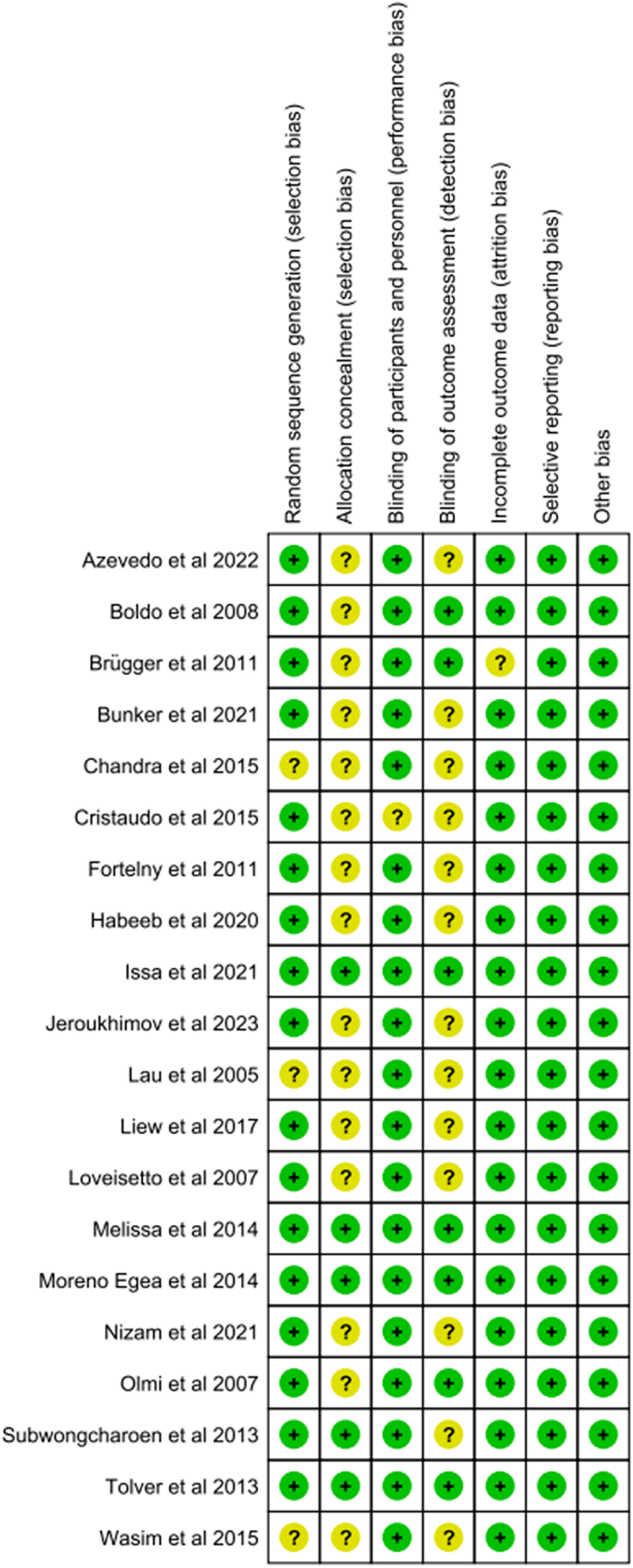
Risk of bias summary.

We assessed the risk of bias using the Cochrane Handbook for Systematic Reviews of Interventions. All the studies included in this meta-analysis were of high quality (Figure 2, 3). The details of the risk assessment are provided in Supplementary Table S2.

### Meta-analysis outcomes

#### Primary outcomes

##### Chronic pain

**Figure 4:**
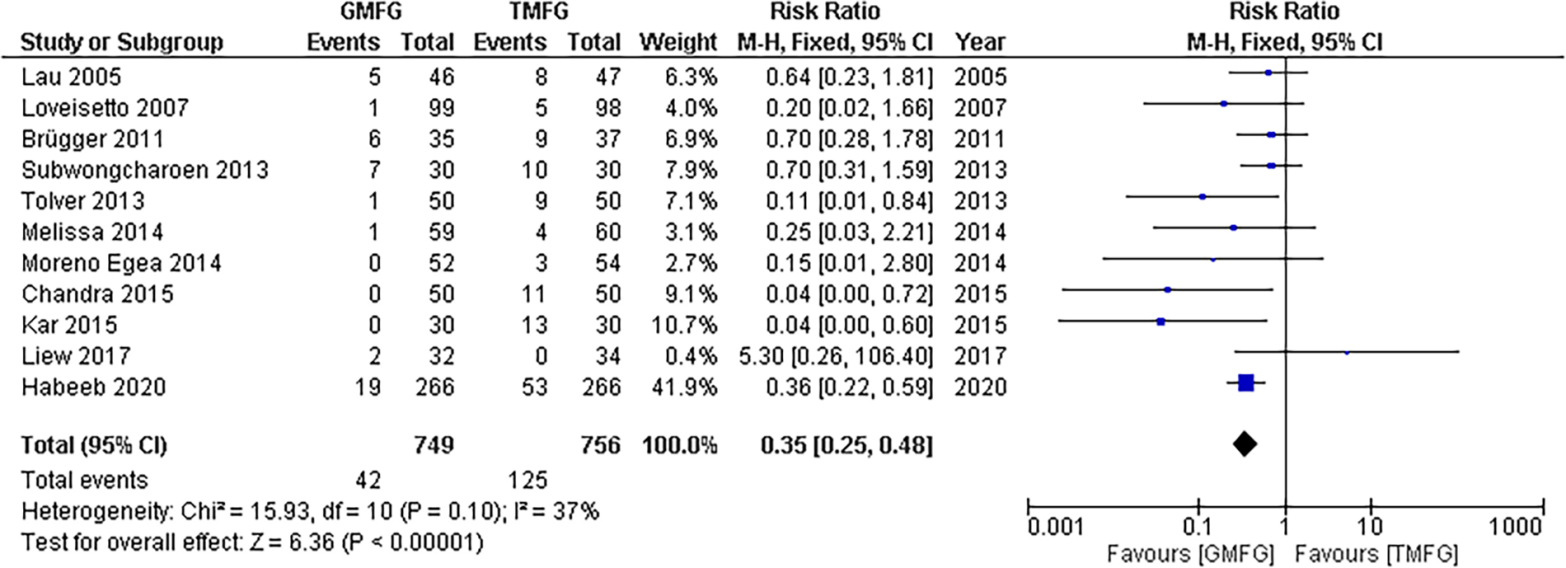
Forest plot of rate of chronic pain.

A meta-analysis using a random-effects model conducted on a total of 11 studies (33, 35, 37, 39, 41, 43–46, 48, 49) consisting of 1,505 patients showed that GMF significantly reduced the incidence of chronic pain in patients who underwent laparoscopic inguinal hernia repair, in comparison with TMF [RR:0.40, 95% CI (0.23,0.68); *p* = 0.0007] (Figure 4). The studies demonstrated a remarkable consistency, unveiling a notable absence of statistical heterogeneity (*p* = 0.10; *I*^2 ^=^ ^37%).

### Pain score on postoperative day 1

**Figure 5:**
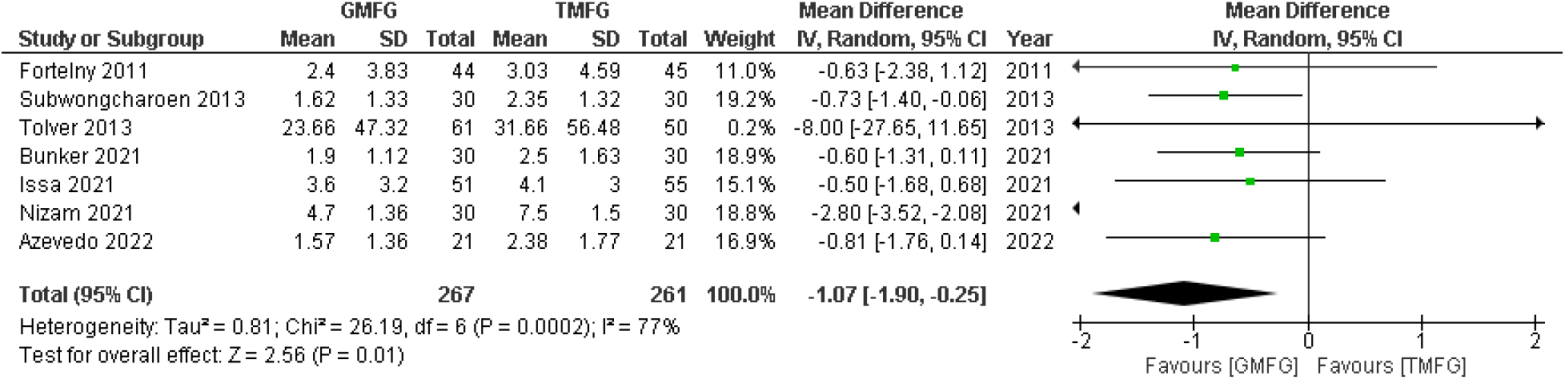
Forest plot of rate of pain score on postoperative day 1.

Seven studies (17, 19, 33, 38, 40, 42, 48), consisting of 528 patients, reported pain scores on postoperative day 1. A random-effects model was used to pool the results, which showed that GMF was significantly associated with decreasing pain score on postoperative day 1 compared with the TMF [MD = −1.07, 95% CI (−1.90,−0.25), *p* = 0.01] (Figure 5). The analysis showed significant heterogeneity among the studies (*p* = 0.0002; *I*^2 ^=^ ^77%).

### Leave-one-out analysis

The leave-one-out sensitivity analysis showed that the pain score on postoperative day 1 was affected by a single study, i.e., Nizam et al. (17). Removing that study resulted in a significant reduction in *I*^2^ values (*p* = 0.98; *I*^2^ = 0%) and overall effect [MD = −0.68, 95% CI (−1.07, −0.28), *p* = 0.0008] (Supplementary Figure S1).

### Secondary outcomes

#### Operation time

**Figure 6:**
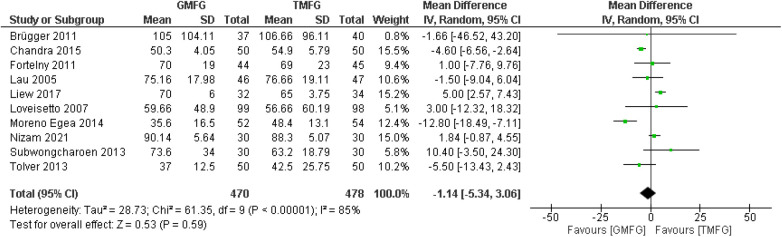
Forest plot of rate of operation time.

A total of 10 studies (17, 33, 35, 37, 39, 40, 43, 44, 46, 48), consisting of 948 patients, provided data on the operation time. A random-effects model was used to pool the combined effect. The results indicated that there was no significant difference in operative time between TMF and GMF [MD = −1.14, 95% CI (−5.34, 3.06), *p* = 0.59] (Figure 6). There was severe heterogeneity among the studies (*p* < 0.00001; *I*^2 ^= 85%).

### Leave-one-out analysis

After systematically removing one study at a time, the results consistently showed that the overall effect was unchanged, which suggests that the results of this study were stable.

### Recurrence rate

**Figure 7:**
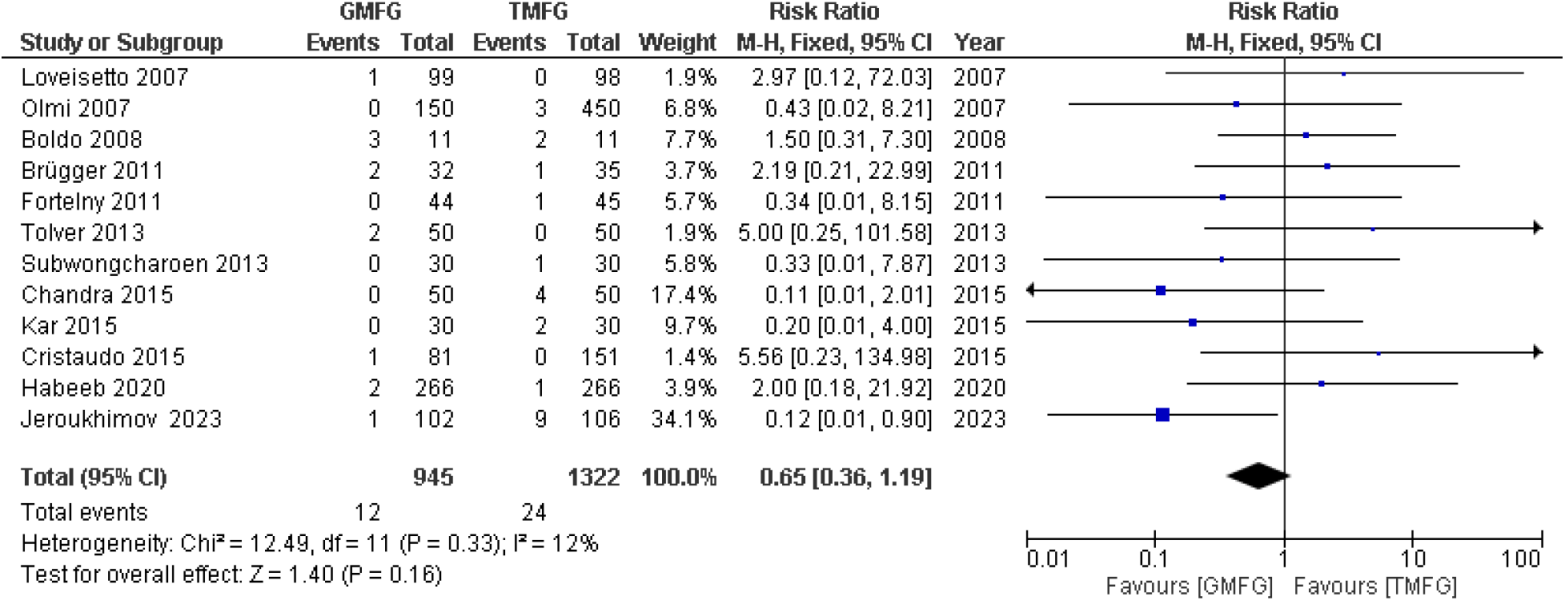
Forest plot of rate of recurrence rate.

A total of 12 studies (15, 33, 34, 36, 37, 39–41, 44, 47–49), consisting of 2,267 patients, reported the incidence of hernia recurrence. A random-effects model was used to pool the combined effect. The studies demonstrated a remarkable consistency, unveiling a notable absence of statistical heterogeneity (*p* = 0.33; *I*^2 ^=^ ^12%). The results for the incidence of recurrence rate showed no significant difference between the two groups [RR = 0.80, 95% CI (0.36, 1.78) *p* = 0.58] (Figure 7).

### Hematoma

**Figure 8:**
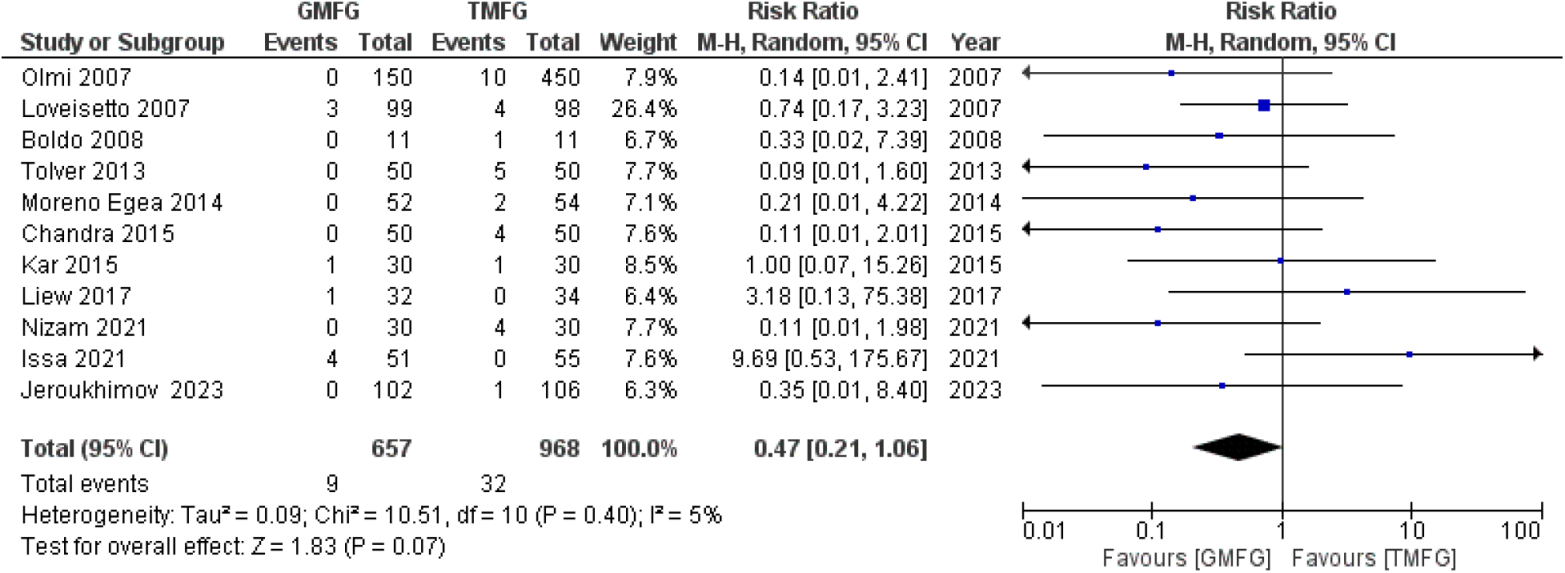
Forest plot of rate of hematoma.

A total of 11 studies (15, 17, 35, 36, 39, 42, 44, 46–49), consisting of 1,625 patients, reported the incidence of hematoma. The incidence of hematoma in the GMF group was 9/657 (1.36%), and it was 32/968 (3.3%) in the TMF group. The studies demonstrated a remarkable consistency, unveiling a notable absence of statistical heterogeneity (*p* = 0.40, *I*^2 ^=^ ^5%). A random-effects model was used to pool the combined effect. The results did not show a significant difference between patients in the GMF group and those in the TMF group [RR: 0.47, 95% CI (0.21, 1.06); *p* = 0.07] (Figure 8).

### Seroma

**Figure 9:**
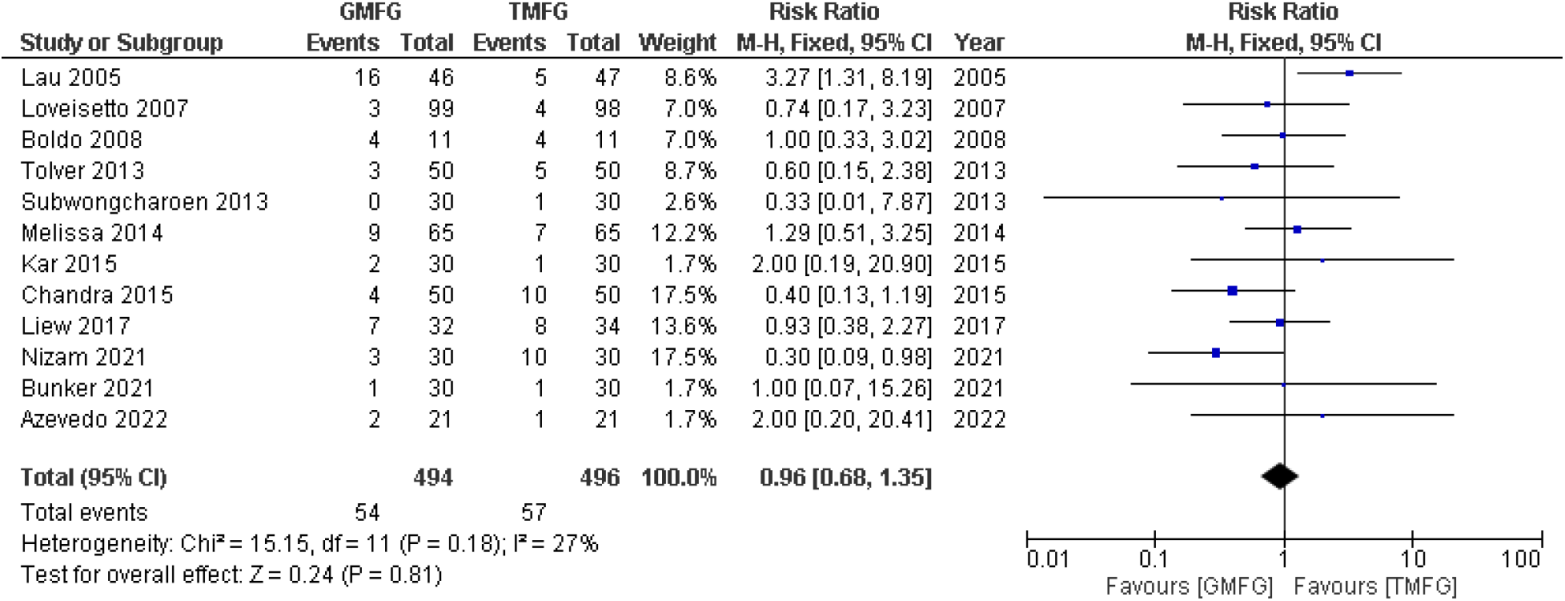
Forest plot of rate of seroma.

A total of 12 studies (17, 19, 33, 35, 36, 38, 39, 43, 44, 45, 48, 49), consisting of 990 patients, reported the occurrence of seroma. The incidence of seroma in the GMF group was 54/494 (10.9%), and the incidence of seroma in the TMF group was 57/496 (11.4%). The studies demonstrated a remarkable consistency, unveiling a notable absence of statistical heterogeneity (*p* = 0.18, *I*^2 ^=^ ^27%). A random-effects model was used to pool the combined effect, which showed that no significant difference was observed between both groups [RR: 0.93, 95% CI (0.59, 1.46); *p* = 0.75] (Figure 9).

### Total complications

**Figure 10:**
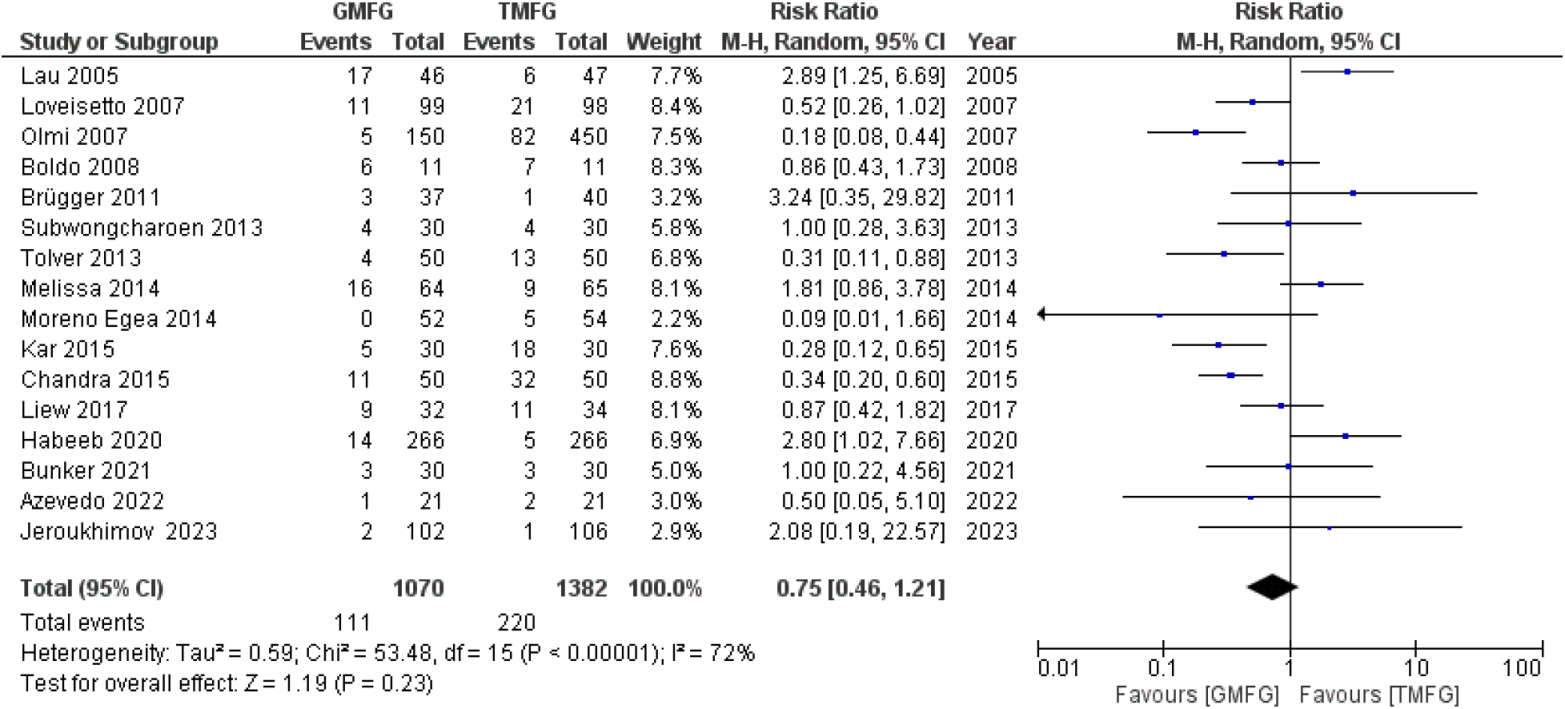
Forest plot of rate of total complications.

A total of 16 studies (15, 19, 33, 35–39, 41, 43–49), consisting of 2,452 patients, reported the incidence of total complications. There was severe statistical heterogeneity found among the studies (*p* < 0.00001, *I*^2 ^=^ ^72%). A random-effects model was used to pool the combined effect. The results did not show any significant difference between the two groups [RR: 0.75, 95% CI (0.46, 1.21); *p* = 0.23] (Figure 10).

### Leave-one-out analysis

To identify the source of significant heterogeneity, a sensitivity analysis was conducted. By systematically excluding studies one by one, it was determined that the results remained unchanged, indicating the robustness and stability of this study's findings.

### Publication bias

To assess publication bias, we employed various methods, including funnel plots and Egger's regression. Six funnel plots were used for the outcomes of chronic pain, operation time, recurrence rate, hematoma, seroma, and total complications (Supplementary Figures S2–S7). The funnel plots displayed a symmetrical distribution of studies, indicating a lack of asymmetry and no indication of publication bias. In addition, statistical tests using Egger's regression did not yield significant results, further supporting the absence of publication bias in our analysis. Egger's test was performed for chronic pain (*p*-value = 0.31), operative time (*p*-value = 0.44), recurrence rate (*p*-value = 0.82), hematoma (*p*-value = 0.58), seroma (*p*-value = 0.68), and total complications (*p*-value = 0.74).

## Discussion

In this meta-analysis, we compared the usage of GMF and TMF in laparoscopic inguinal hernia repair. We concluded that GMF is significantly associated with a lower incidence of chronic pain and postoperative pain score on day 1. However, there was no evidence to suggest that GMF reduces operation time, hematoma, recurrence rate, total complications, and seroma. In laparoscopic inguinal hernia surgery, three primary techniques are employed for securing the mesh. Of these, the application of suture fixation patches is intricate, time-intensive, and infrequently implemented (50). Currently, the prevailing approach in clinical settings involves the frequent utilization of glue or tacks for mesh fixation. The EHS classification system (51), akin to its predecessors, lacks a formal Delphi methodology and a rigorous validity evaluation, which leads to a weak recommendation in the HerniaSurge guidelines (52) for research purposes. It was not employed in the source studies reviewed here, and therefore, the impact of hernia classification on chronic pain and other outcomes was not assessed, aligning with the non-objective of this pragmatic review of RCTs (53).

Postoperative pain following inguinal hernia repair is a prevalent occurrence. The outcomes of our meta-analysis indicated a noteworthy decrease in the prevalence of persistent pain within the GMF group (5.6%) in comparison with the TMF group (16.5%), aligning with the findings of a prior meta-analysis conducted by Nan Hu (20). Numerous studies propose that chronic pain often stems from factors such as nerve traction injury, suture-related issues, mesh interaction, scar tissue compression, and injuries to the pubic tubercle periosteum and spermatic cord (54). Opting for GMF mitigates the risk of nerve damage and compression, while also sparing the periosteum from harm, thereby resulting in a substantial reduction in the incidence of chronic pain (55). In a similar vein, this meta-analysis revealed a statistically significant distinction in pain scores on the first day post-surgery, indicating that GMF resulted in a lower score compared with TMF. Notably, this discovery contradicts the outcomes reported in an earlier meta-analysis (20). In the GMF, the glue was applied to secure the mesh, yet the disparity in operation time between the two groups lacked statistical significance. This suggests that incorporating a glue-fixed mesh in laparoscopic inguinal hernia surgery is unlikely to markedly prolong the procedure, and it remains a straightforward process. Similar outcomes were observed in a previous analysis (20). Local hematoma is a frequent complication in inguinal hernia surgery, often stemming from vascular injury. In laparoscopic procedures, it demands careful attention as it can escalate into a sizable retroperitoneal hematoma, potentially requiring reoperation in unstable patients (56). The higher incidence of hematoma in the TMF may be attributed to injuries in the peritoneum or small muscle vessels, whereas the GMF, steering clear of tissue trauma, displayed a lower occurrence of hematoma (40, 57). However, our analysis indicates a lower hematoma incidence in GMF (1.36%) compared with TMF (3.3%), although this difference lacks statistical significance. Due to the limited sample size, future studies with a larger sample size are needed to thoroughly investigate potential differences in hematoma incidence between GMF and TMF. An RCT conducted by Lau et al. (43) has determined that the occurrence of seroma formation is elevated in the GMF group. This phenomenon has been ascribed to a more pronounced inflammatory response prompted by fibrin glue, potentially amplifying exudation and, consequently, the development of seromas (58). However, our analysis did not reveal a statistically significant difference between the two groups, aligning with the conclusions drawn from previous meta-analyses (55, 57). The recurrence rate determines the success rate of inguinal hernia repair. The rate of hernia recurrence in this meta-analysis was 1.26% in the GMF group and 1.81% in the TMF group. The lack of a statistically significant difference between the two groups implies that utilizing glue for mesh fixation does not enhance the risk of hernia recurrence. These results are consistent with the conclusions reached in earlier meta-analyses (20, 57). This meta-analysis also indicates that the GMF had a lower overall complication rate of 10.37% when compared with the TMF, which has a rate of 15.91%. This reduction, however, is not statistically significant, showing that employing adhesive in laparoscopic tension-free inguinal hernia repair is still a safe option. These findings contradict the conclusions of a study conducted by Nan et al. (20), which asserted that GMF is linked to a lower occurrence of overall complications compared with TMF. This disparity could be explained by the omission of several studies that reported overall complication rates for both groups. Furthermore, the use of fixed-effects models for analysis rather than random-effects models, as advised when dealing with studies of varied sizes, may have contributed to the inconsistency of the results (21).

The limitations identified in this meta-analysis encompass (1) the inclusion of a small number of studies with limited sample sizes; (2) potential language bias due to the restriction to English literature; (3) the inevitable impact on the results from variations in techniques, procedures, mesh materials and types, glue compositions, and mechanical fixation materials across studies; (4) the unavailability of relevant data on cost considerations, preventing an analysis on cost-effectiveness to guide a preference for either method; (5) some studies overlooking the importance of randomization, double-blinding, and allocation concealment in randomized controlled trials, thus influencing the strength of evidence; and (6) inconsistency in follow-up durations among studies, with some lacking sufficient short-term follow-up to adequately assess and compare recurrence rates between the two groups.

In conclusion, this comprehensive meta-analysis compared GMF and TMF in laparoscopic inguinal hernia repair. GMF demonstrated a significant reduction in chronic pain incidence and postoperative pain scores on the first day compared with TMF. Operation time, recurrence rate, hematoma, seroma, and overall complication rates showed no significant differences between the two methods. The recurrence rate was notably low in both groups, with GMF exhibiting a slightly lower overall complication rate. Despite some contradictions with previous meta-analyses, our analysis emphasizes the safety and efficacy of both fixation methods. Limitations include the small number of studies, potential language bias, variations in techniques, and the absence of cost-effectiveness analysis due to data unavailability. Future research with larger sample sizes and comprehensive considerations is warranted.

## Data Availability

The original contributions presented in the study are included in the article/Supplementary Material, further inquiries can be directed to the corresponding author.
