# Supplementary material for: Safety and efficacy revisited: a systematic review and meta-analysis of glue versus tack mesh fixation in laparoscopic inguinal herniorrhaphy
Source: Front Surg. 2024 Feb 9;11:1321325. doi: 10.3389/fsurg.2024.1321325 (PMC10884233; doi:10.3389/fsurg.2024.1321325)
Supplement: Supplementary file 1 [file Datasheet1.docx]

**Supplementary Material**

**Supplementary Figure 1**. Forest plot of rate of Pain Score on Post-Op Day 1


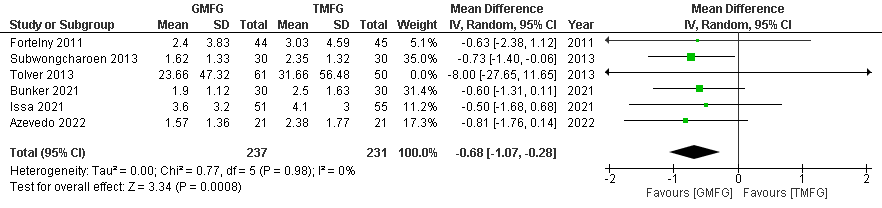


**Supplementary Figure 2**. Funnel plot of Chronic Pain

**
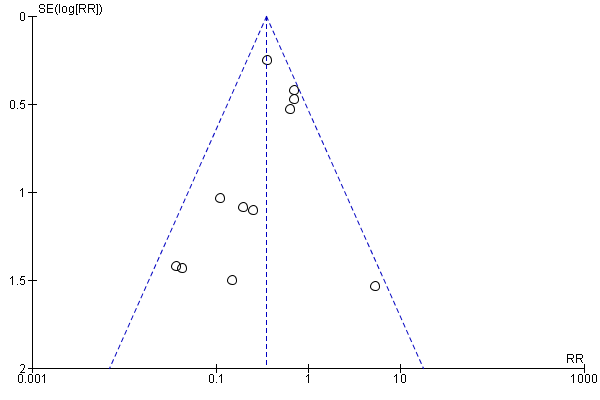
**

**Supplementary Figure 3**. Funnel plot of Operation Time


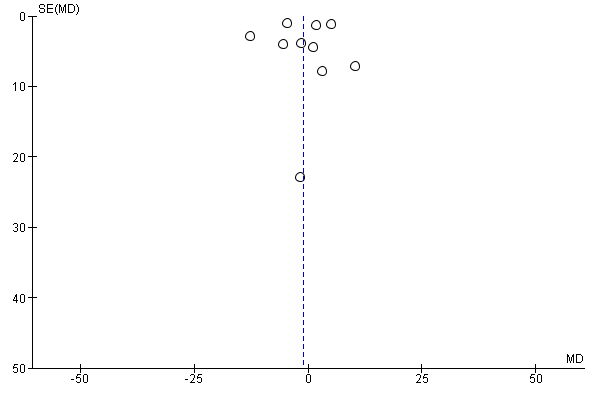


**Supplementary Figure 4**. Funnel plot of Recurrence Rate


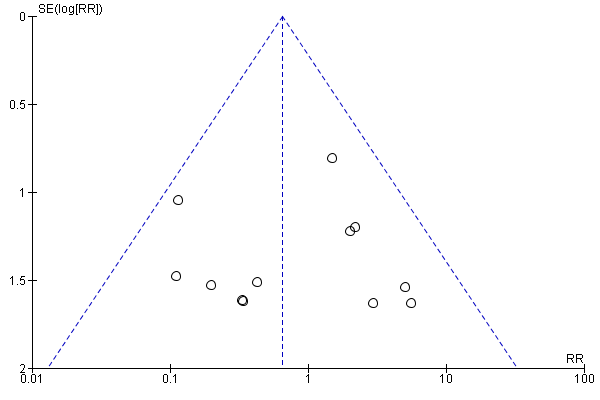


**Supplementary Figure 5**. Funnel plot of Hematoma


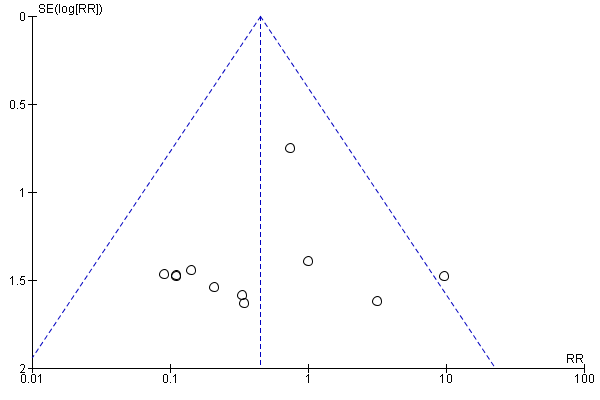


**Supplementary Figure 6**. Funnel plot of Seroma


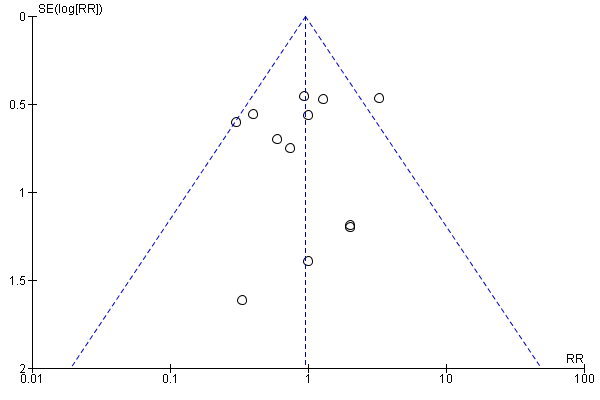


**Supplementary Figure 7**. Funnel plot of Total Complications

**
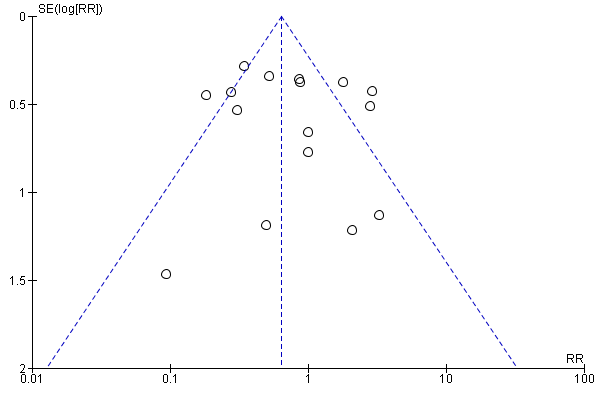
**


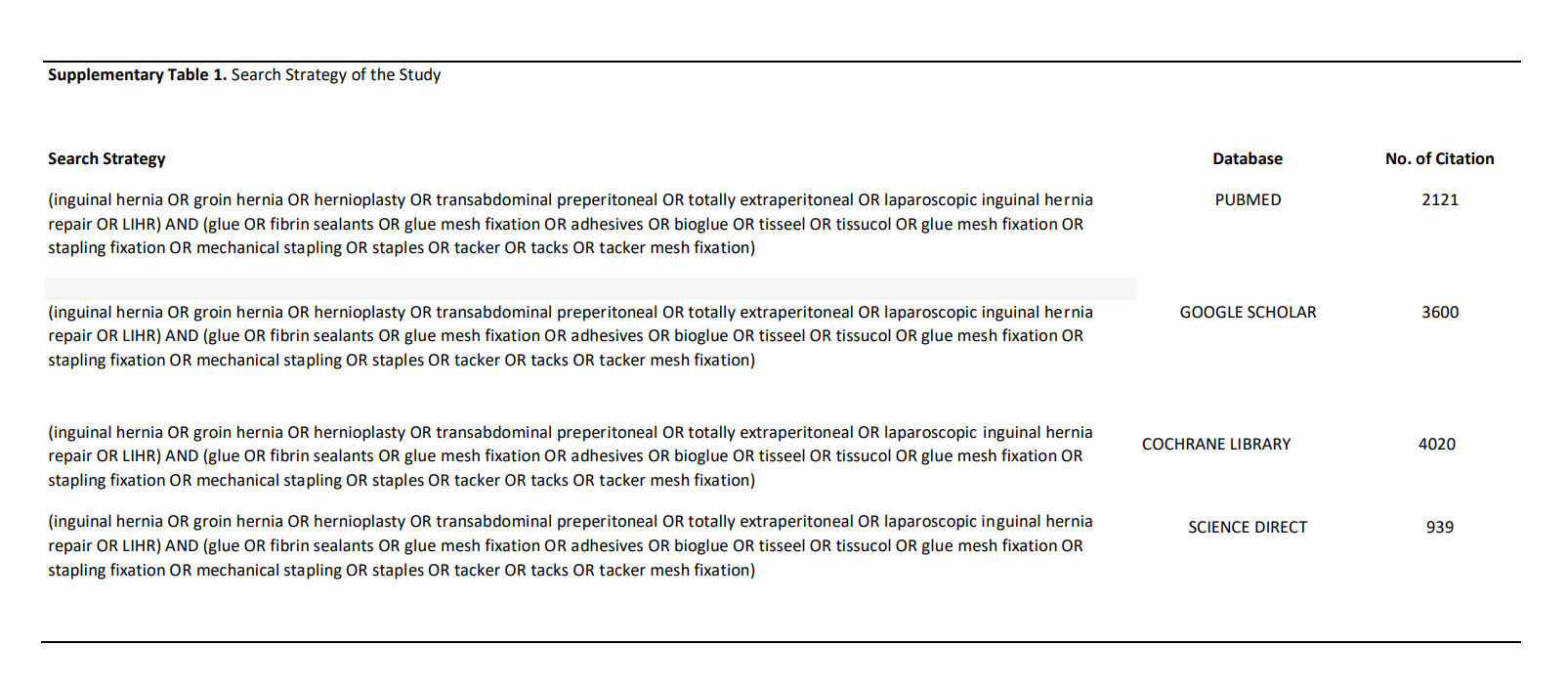


**Supplementary Table 2**. Risk of Bias Assessment Table with Reasons

|  | Cochrane Risk-of-Bias Tool | | |
| --- | --- | --- | --- |
|  | Bias | Risk of bias | Author judgement |
| BOLDO 2008 | Random sequence generation (selection bias) | Low Risk | Quote: “Using a randomization table, random assignment of each side to either mesh fixation with fibrin (FG) or with staples (SG) was performed preoperatively.” |
|  | Allocation concealment (selection bias) | Unclear Risk | Quote: “Insufficient detail to allow definitive judgement.” |
|  | Blinding of participants and personnel (performance bias) | Low Risk | Quote: “Unicenter single surgeon prospective randomized double-blind study of transabdominal preperitoneal (TAPP) bilateral hernioplasties comparing autologous fibrin sealant (FG) used for mesh fixation on one side and staples (SG) on the other.” |
|  | Blinding of outcome assessment (detection bias) | Low Risk | Quote: “This was a double-blind study. Neither the patient nor the surgeon (G.P.d.L.) in charge of collecting the postoperative evaluation had knowledge of the method of mesh fixation used on either side.” |
|  | Incomplete outcome data (attrition bias) | Low Risk | Quote: “No missing outcome data.” |
|  | Selective reporting (reporting bias) | Low Risk | All pre-specified endpoints were reported. |
|  | Other bias | Low Risk | The study appears to be free of other sources of bias. |
| BRUGGERS 2011 | Random sequence generation (selection bias) | Low Risk | Quote: “Randomization in permutated blocks of 20 was performed with sealed envelopes. The random distribution between the two groups was assessed with www.randomiza tion.com.” |
|  | Allocation concealment (selection bias) | Unclear Risk | Quote: “Insufficient detail to allow definitive judgement.” |
|  | Blinding of participants and personnel (performance bias) | Low Risk | Quote: “Patients were blinded to the method of mesh fixation (single blinded).” |
|  | Blinding of outcome assessment (detection bias) | Low Risk | Quote: “Follow-up after discharge included physical examination and an interview by an independent observer. |
|  | Incomplete outcome data (attrition bias) | Unclear Risk | Quote: “Insufficient detail to allow definitive judgement.” |
|  | Selective reporting (reporting bias) | Low Risk | Quote: “All pre-specified endpoints were reported.” |
|  | Other bias | Low Risk | Quote: “The study appears to be free of other sources of bias.” |
| BUNKER 2021 | Random sequence generation (selection bias) | Low Risk | Quote: Randomization was done using sealed envelopes with random numbers in the operating theatre.” |
|  | Allocation concealment (selection bias) | Unclear Risk | Quote: “Insufficient detail to allow definitive judgement.” |
|  | Blinding of participants and personnel (performance bias) | Low Risk | Quote: “Patients were blinded about group allocation..” |
|  | Blinding of outcome assessment (detection bias) | Unclear Risk | Quote: “Insufficient detail to allow definitive judgement.” |
|  | Incomplete outcome data (attrition bias) | Low Risk | Quote: “No missing outcome data.” |
|  | Selective reporting (reporting bias) | Low Risk | Quote: “All pre-specified endpoints were reported.” |
|  | Other bias | Low Risk | Quote: “The study appears to be free of other sources of bias.” |
| CHANDRA 2015 | Random sequence generation (selection bias) | Unclear Risk | Quote: “Insufficient detail to allow definitive judgement.” |
|  | Allocation concealment (selection bias) | Unclear Risk | Quote: “Insufficient detail to allow definitive judgement.” |
|  | Blinding of participants and personnel (performance bias) | Low Risk | Quote: “All 100 patients were divided into 50 blocks (block randomisation). Within a block, one patient was case while other was control. For randomisation, randomiser software from www.randomizer.com was used.’ |
|  | Blinding of outcome assessment (detection bias) | Unclear Risk | Quote: “Insufficient detail to allow definitive judgement.” |
|  | Incomplete outcome data (attrition bias) | Low Risk | Quote: “No missing outcome data.” |
|  | Selective reporting (reporting bias) | Low Risk | All pre-specified endpoints were reported. |
|  | Other bias | Low Risk | The study appears to be free of other sources of bias. |
| CRISTAUDO 2015 | Random sequence generation (selection bias) | Low Risk | patients were prospectively randomised |
|  | Allocation concealment (selection bias) | Unclear Risk | Quote: “Insufficient detail to allow definitive judgement.” |
|  | Blinding of participants and personnel (performance bias) | Unclear Risk | Quote: “Insufficient detail to allow definitive judgement.” |
|  | Blinding of outcome assessment (detection bias) | Unclear Risk | Quote: “Insufficient detail to allow definitive judgement.” |
|  | Incomplete outcome data (attrition bias) | low Risk | Quote: “No missing outcome data.” |
|  | Selective reporting (reporting bias) | Low Risk | All pre-specified endpoints were reported. |
|  | Other bias | Low Risk | The study appears to be free of other sources of bias. |
| FORTELNY 2011 | Random sequence generation (selection bias) | Low Risk | Quote: “, the randomization was performed using a web-based program provided by the Department of Statistics of the Vienna Medical School. Patients were randomized into group A or group B.” |
|  | Allocation concealment (selection bias) | Unclear Risk | Quote: “Insufficient detail to allow definitive judgement.” |
|  | Blinding of participants and personnel (performance bias) | Low Risk | Quote: “male patients were recruited in the outpatient ward of our department. These patients underwent a standard TAPP procedure [17] by a single surgeon” |
|  | Blinding of outcome assessment (detection bias) | Unclear Risk | Quote: “Insufficient detail to allow definitive judgement.” |
|  | Incomplete outcome data (attrition bias) | Low Risk | Quote: “No missing outcome data.” |
|  | Selective reporting (reporting bias) | Low Risk | All pre-specified endpoints were reported. |
|  | Other bias | Low Risk | The study appears to be free of other sources of bias. |
| HABEEB 2020 | Random sequence generation (selection bias) | Low Risk | Quote: “1 Patients were randomly allocated using a random sequence generator.” |
|  | Allocation concealment (selection bias) | Unclear Risk | Quote: “Insufficient detail to allow definitive judgement.” |
|  | Blinding of participants and personnel (performance bias) | Low Risk | Quote: “Random allocations were sequentially numbered in sealed opaque envelopes, which were opened during surgery before carrying out the method of fixation. Patients were blinded to the assigned group until after the study.” |
|  | Blinding of outcome assessment (detection bias) | Unclear Risk | Quote: “Insufficient detail to allow definitive judgement.” |
|  | Incomplete outcome data (attrition bias) | Low Risk | Quote: “No missing outcome data.” |
|  | Selective reporting (reporting bias) | Low Risk | All pre-specified endpoints were reported. |
|  | Other bias | Low Risk | The study appears to be free of other sources of bias. |
| ISSA 2021 | Random sequence generation (selection bias) | Low Risk | Quote: “For allocation of patients into each group, random sequence generation was performed through the use of a study envelope which contained questionnaires for either glue or tack fixation in a 1:1 ratio. Simple randomization was performed by selecting a questionnaire from this envelope on the day of the patient’s surgery, once they were enrolled into the trial. This provided random allocation of the patient to 1 group of the tria.” |
|  | Allocation concealment (selection bias) | Low Risk | Quote: “The envelope was opaque, thus ensuring appropriate allocation concealment until after the questionnaire had been pulled from the envelope. The study envelope was stored within a secured office. The sequence generation and allocation concealment processes were undertaken by the chief investigator of the trial.” |
|  | Blinding of participants and personnel (performance bias) | Low Risk | Quote: “The envelope was opaque, thus ensuring appropriate allocation concealment until after the questionnaire had been pulled from the envelope.” |
|  | Blinding of outcome assessment (detection bias) | Low Risk | Quote: “All outcomes were assessed by a single investigator, and reviewed by the investigating team, during the entirety of the study.” |
|  | Incomplete outcome data (attrition bias) | Low Risk | Quote: “No missing outcome data.” |
|  | Selective reporting (reporting bias) | Low Risk | All pre-specified endpoints were reported. |
|  | Other bias | Low Risk | The study appears to be free of other sources of bias. |
| LAU 2005 | Random sequence generation (selection bias) | Unclear Risk | Quote: “Insufficient detail to allow definitive judgement.” |
|  | Allocation concealment (selection bias) | Unclear Risk | Quote: “Insufficient detail to allow definitive judgement.” |
|  | Blinding of participants and personnel (performance bias) | Low Risk | Quote: “. Eligible patients were randomized to 2 arms of treatment, FS group and staple group, by sealed envelopes containing random number in the operation theater.” |
|  | Blinding of outcome assessment (detection bias) | Unclear Risk | Quote: “Insufficient detail to allow definitive judgement.” |
|  | Incomplete outcome data (attrition bias) | Low Risk | Quote: “No missing outcome data.” |
|  | Selective reporting (reporting bias) | Low Risk | All pre-specified endpoints were reported. |
|  | Other bias | Low Risk | The study appears to be free of other sources of bias. |
| LIEW 2017 | Random sequence generation (selection bias) | Low Risk | Quote: “Randomization was done using serially numbered sealed envelopes with random numbers in the operating theater.” |
|  | Allocation concealment (selection bias) | Unclear Risk | Quote: “Insufficient detail to allow definitive judgement.” |
|  | Blinding of participants and personnel (performance bias) | Low Risk | Quote: “This single center, single blinded, interventional and prospective randomized trial involving patients with unilateral uncomplicated inguinal hernia subjected to undergo TEP repair in Ipoh General Hospital, Malaysia.” |
|  | Blinding of outcome assessment (detection bias) | Unclear Risk | Quote: “Insufficient detail to allow definitive judgement.” |
|  | Incomplete outcome data (attrition bias) | Low Risk | Quote: “No missing outcome data.” |
|  | Selective reporting (reporting bias) | Low Risk | All pre-specified endpoints were reported. |
|  | Other bias | Low Risk | The study appears to be free of other sources of bias. |
| LOVEISETTO 2007 | Random sequence generation (selection bias) | Low Risk | Quote: “Randomization was carried out using randomization tables. In patients with bilateral hernias, both sides were repaired simultaneously; one side was chosen randomly to be the “study hernia” to be included in the intention-to-treat analysis.” |
|  | Allocation concealment (selection bias) | Unclear Risk | Quote: “Insufficient detail to allow definitive judgement.” |
|  | Blinding of participants and personnel (performance bias) | Low Risk | Quote: “Patients were not aware of their treatment group prior to surgery. During the consent process prior to the trial, patients were not informed of our hypothesis that fibrin glue fixation results in less postoperative pain and neuralgia than fixation with staples.” |
|  | Blinding of outcome assessment (detection bias) | Unclear Risk | Quote: “Insufficient detail to allow definitive judgement.” |
|  | Incomplete outcome data (attrition bias) | Low Risk | Quote: “No missing outcome data.” |
|  | Selective reporting (reporting bias) | Low Risk | Quote: “All pre-specified endpoints were reported.” |
|  | Other bias | Low Risk | Quote: “The study appears to be free of other sources of bias.” |
| MELISSA 2014 | Random sequence generation (selection bias) | Low Risk | Quote: “.Patients were randomized after complete dissection of the posterior wall of the inguinal canal. They were randomly allocated to the FS spray (FS group) or MS (MS group) for mesh fixation.” |
|  | Allocation concealment (selection bias) | Low Risk | Quote: “allocations were placed in consecutively numbered and sealed, opaque envelopes.” |
|  | Blinding of participants and personnel (performance bias) | Low Risk | Quote: “This was a prospective double-blinded randomized controlled trial performed at the Ambulatory Surgery Center of Alice Ho Miu Ling Nethersole Hospital in Hong Kong. B.” |
|  | Blinding of outcome assessment (detection bias) | Low Risk | Quote: “All outcome assessments were performed by a designated nurse of the Ambulatory Surgery Center who was blinded to the type of mesh fixation.” |
|  | Incomplete outcome data (attrition bias) | Low Risk | Quote: “No missing outcome data.” |
|  | Selective reporting (reporting bias) | Low Risk | Quote: “All pre-specified endpoints were reported.” |
|  | Other bias | Low Risk | Quote: “The study appears to be free of other sources of bias.” |
| MORENO EGEA 2014 | Random sequence generation (selection bias) | Low Risk | Quote: “Randomization was achieved by a computer program, and all patients were blinded to the allocation.” |
|  | Allocation concealment (selection bias) | Low Risk | Quote” and all patients were blinded to the allocation” |
|  | Blinding of participants and personnel (performance bias) | Low Risk | Quote: “This randomized, single-blind trial was conducted in the Ambulatory Abdominal Wall Unit of Morales Meseguer University Hospital in Murcia, Spain. The study enrolment took place between January 2008 and January 2011..” |
|  | Blinding of outcome assessment (detection bias) | Low Risk | Quote: “Comparisons of dichotomous outcomes were made using Pearson’s χ2 test, and an analysis of smaller groups within the study was permitted, using Fisher’s exact test. P < .05 was considered significant.” |
|  | Incomplete outcome data (attrition bias) | Low Risk | Quote: “No missing outcome data.” |
|  | Selective reporting (reporting bias) | Low Risk | All pre-specified endpoints were reported. |
|  | Other bias | Low Risk | The study appears to be free of other sources of bias. |
| NIZAM 2021 | Random sequence generation (selection bias) | Low Risk | Quote: “Patients were randomized into two groups by opaque sealed envelope method before fixation of the mesh during surgery into two groups—group A and group B.” |
|  | Allocation concealment (selection bias) | Unclear Risk | Quote: “Insufficient detail to allow definitive judgement.” |
|  | Blinding of participants and personnel (performance bias) | Low Risk | Quote: “A randomized controlled single blinded study was conducted “ and “The opaque sealed envelope was opened and patients were randomized into the respective groups.” |
|  | Blinding of outcome assessment (detection bias) | Unclear Risk | Quote: “Insufficient detail to allow definitive judgement.” |
|  | Incomplete outcome data (attrition bias) | Low Risk | Quote: “No missing outcome data.” |
|  | Selective reporting (reporting bias) | Low Risk | All pre-specified endpoints were reported. |
|  | Other bias | Low Risk | The study appears to be free of other sources of bias. |
| OLMI 2007 | Random sequence generation (selection bias) | Low Risk | Quote: “. The type of procedure was randomized.” |
|  | Allocation concealment (selection bias) | Unclear Risk | Quote: “Insufficient detail to allow definitive judgement.” |
|  | Blinding of participants and personnel (performance bias) | Low Risk | Quote: “follow-up visits were conducted by an additional surgeon blinded, as well as the patient, to what sort of fixation was used.” |
|  | Blinding of outcome assessment (detection bias) | Low Risk | Quote: “follow-up visits were conducted by an additional surgeon blinded, as well as the patient, to what sort of fixation was used. These interviews incorporated assessment for pain quantified using a VAS and complications including hematoma, seroma, urinary retention, and recurrences..” |
|  | Incomplete outcome data (attrition bias) | Low Risk | Quote: “No missing outcome data.” |
|  | Selective reporting (reporting bias) | Low Risk | All pre-specified endpoints were reported. |
|  | Other bias | Low Risk | The study appears to be free of other sources of bias. |
| SUBWONGCHAROEN 2013 | Random sequence generation (selection bias) | LowRisk | by block randomization |
|  | Allocation concealment (selection bias) | low Risk | The anesthesiologist was blinded to the type of fixation |
|  | Blinding of participants and personnel (performance bias) | low Risk | Patients, assessor, and anesthetic nurse were blinded to randomization |
|  | Blinding of outcome assessment (detection bias) | Unclear Risk | Quote: “Insufficient detail to allow definitive judgement |
|  | Incomplete outcome data (attrition bias) | Low Risk | Quote: “No missing outcome data.” |
|  | Selective reporting (reporting bias) | Low Risk | All pre-specified endpoints were reported. |
|  | Other bias | Low Risk | The study appears to be free of other sources of bias. |
| TOLVER 2013 | Random sequence generation (selection bias) | Low Risk | Quote: “We used block randomization based on computer-generated sequences with block size of 4 (112 consecutive sealed non-transparent envelopes numbered 1–112).” |
|  | Allocation concealment (selection bias) | Low Risk | Quote: “The undisclosed code A or B was used in the patient records. The randomization code was concealed until the study was finished and data analyses completed” |
|  | Blinding of participants and personnel (performance bias) | Low Risk | Quote: “On the day of operation, the surgeon opened an envelope just before the operation. The surgeon, the anaesthetist and the nurses at the operating theatre were for obvious reasons not blinded, while the patient, the investigator and nurses attending to the patient postoperatively were all blinded to the intervention. |
|  | Blinding of outcome assessment (detection bias) | Low Risk | Quote: “the patient, the investigator and nurses attending to the patient postoperatively were all blinded to the intervention..” |
|  | Incomplete outcome data (attrition bias) | Low Risk | Quote: “No missing outcome data.” |
|  | Selective reporting (reporting bias) | Low Risk | All pre-specified endpoints were reported. |
|  | Other bias | Low Risk | The study appears to be free of other sources of bias. |
| WASIM 2015 | Random sequence generation (selection bias) | Unclear Risk | Quote: “Insufficient detail to allow definitive judgement.” |
|  | Allocation concealment (selection bias) | Unclear Risk | Quote: “Insufficient detail to allow definitive judgement.” |
|  | Blinding of participants and personnel (performance bias) | Low Risk | Quote: “No attempt was made at formal blinding of patients or caregivers following concealed randomization .” |
|  | Blinding of outcome assessment (detection bias) | Unclear Risk | Quote: “Insufficient detail to allow definitive judgement.” |
|  | Incomplete outcome data (attrition bias) | Low Risk | Quote: “No missing outcome data.” |
|  | Selective reporting (reporting bias) | Low Risk | All pre-specified endpoints were reported. |
|  | Other bias | Low Risk | The study appears to be free of other sources of bias. |
| AZEVEDO 2022 | Random sequence generation (selection bias) | Low Risk | a randomization application was used to allocate them into three different groups as mesh-fixation groups. |
|  | Allocation concealment (selection bias) | Unclear Risk | Quote: “Insufficient detail to allow definitive judgement.” |
|  | Blinding of participants and personnel (performance bias) | low Risk | patients were evaluated through physical examination by a surgeon who had not participated in the surgery |
|  | Blinding of outcome assessment (detection bias) | Unclear Risk | Quote: “Insufficient detail to allow definitive judgement.” |
|  | Incomplete outcome data (attrition bias) | Low Risk | Quote: “No missing outcome data |
|  | Selective reporting (reporting bias) | Low Risk | All pre-specified endpoints were reported. |
|  | Other bias | Low Risk | The study appears to be free of other sources of bias. |
| JEROUKHIMOV 2023 | Random sequence generation (selection bias) | Low Risk | Randomization was made using sealed envelopes, which were opened in the operating room just before mesh fixation |
|  | Allocation concealment (selection bias) | Unclear risk | Quote: “Insufficient detail to allow definitive judgement |
|  | Blinding of participants and personnel (performance bias) | low Risk | The examinations and telephone interviews were performed by an attending surgeon blinded to the method of mesh fixation.” |
|  | Blinding of outcome assessment (detection bias) | Unclear Risk | Quote: “Insufficient detail |
|  | Incomplete outcome data (attrition bias) | Low Risk | Quote: “No missing outcome data.” |
|  | Selective reporting (reporting bias) | Low Risk | All pre-specified endpoints were reported. |
|  | Other bias | Low Risk | The study appears to be free of other sources of bias. |
